# Photoacoustic Imaging in Biomedicine and Life Sciences

**DOI:** 10.3390/life12040588

**Published:** 2022-04-14

**Authors:** Alexey Neprokin, Christian Broadway, Teemu Myllylä, Alexander Bykov, Igor Meglinski

**Affiliations:** 1 Opto-Electronics and Measurements, ITEE, University of Oulu, P.O. Box 4500, 90570 Oulu, Finland; aleksei.neprokin@oulu.fi (A.N.); teemu.myllyla@oulu.fi (T.M.); alexander.bykov@oulu.fi (A.B.); 2 College of Engineering and Physical Sciences, Aston University, Birmingham B4 7ET, UK; c.broadway2@aston.ac.uk; 3 Research Unit of Medical Imaging, Physics and Technology, Faculty of Medicine, University of Oulu, P.O. Box 4500, 90014 Oulu, Finland; 4 Department of Histology, Cytology and Embryology, Institute of Clinical Medicine N.V. Sklifosovsky, I.M. Sechenov First Moscow State Medical University, 119435 Moscow, Russia; 5 Interdisciplinary Laboratory of Biophotonics, National Research Tomsk State University, 634050 Tomsk, Russia; 6 REC Fundamental and Applied Photonics, Nanophotonics, Immanuel Kant Baltic Federal University, 236041 Kaliningrad, Russia

**Keywords:** photo-acoustics, opto-acoustic imaging, biomedical imaging, photo-acoustic tomography, photo-acoustic microscopy, photo-acoustic endoscopy

## Abstract

Photo-acoustic imaging, also known as opto-acoustic imaging, has become a widely popular modality for biomedical applications. This hybrid technique possesses the advantages of high optical contrast and high ultrasonic resolution. Due to the distinct optical absorption properties of tissue compartments and main chromophores, photo-acoustics is able to non-invasively observe structural and functional variations within biological tissues including oxygenation and deoxygenation, blood vessels and spatial melanin distribution. The detection of acoustic waves produced by a pulsed laser source yields a high scaling range, from organ level photo-acoustic tomography to sub-cellular or even molecular imaging. This review discusses significant novel technical solutions utilising photo-acoustics and their applications in the fields of biomedicine and life sciences.

## 1. Introduction

Photo-acoustic imaging (PA or PAI) is based upon the generation of rapid thermo-elastic expansion and contraction within soft biological tissues through incident laser pulses. This localised thermal expansion leads to the subsequent emission of ultrasonic waves, which are then reconstructed in two or more dimensions to portray the internal spatial distribution of optical absorption [1,2,3,4,5,6,7,8,9,10,11]. Experiencing much less scattering compared to conventional optical techniques, PAI propagates deeper into biological tissues and provides higher spatial imaging resolution compared to conventional optical imaging techniques for high scattering objects, thereby breaking through the optical diffusion limit ultrasonically [12]. In the last few decades, through intensive study and elaboration, sub-micrometer scale resolution and depths of up to ∼70 mm can be reached. By manipulating acoustic and optical foci, PAI can be set-up for widely different scales. PAI can therefore visualise the internal structures of biological tissues in the organelles to organ range, extending to whole-body imaging for small animals. Targeted at absorbing particles, it is capable of obtaining high contrast from a variety of endogenous and exogenous sources including haemoglobin, melanin, DNA, nano-particles, and chemical dyes etc. [13,14,15]. Structural and functional information such as blood vessel distribution, oxygenation and deoxygenation, tumours, brain functions, lipid concentration and more can be clearly observed [16]. This review highlights the fundamentals and key developments of PAI, discusses the current challenges associated with this technique and focuses on selected major applications in biomedicine and life sciences.

## 2. Fundamentals of Photoacoustic Imaging

### 2.1. PA Signal Generation and Imaging

Thermal and stress confinement are achieved when a laser pulse satisfies the following conditions [17]:

(1) $$t_{0}<\frac{d}{\nu_{s}}<\frac{d^{2}}{4\alpha },$$

where $t_{0}$ is the laser pulse duration, *d* is the depth size of the optical absorption volume (heated zone), $\nu_{s}$ is the acoustic speed in the volume, and α is the thermal diffusion (m${}^{2}$/s). This suggests that within the laser pulse width the induced initial pressure has not travelled beyond the heated zone. Considering heat conduction and fractional volume expansion are negligible [18], the initial pressure $\rho_{0}$ can be defined as:

(2) $$\rho_{0}=\frac{\beta }{k\rho C_{\nu }}\mu_{\alpha }\Psi =\Gamma \mu_{\alpha }\Psi .$$

where, $\mu_{\alpha }$ is the optical absorption coefficient, Ψ is the laser fluency, ρ is the density, $\Gamma =\frac{\beta }{k\rho C_{\nu }}$ is defined as the Grüneisen parameter, β is the thermal expansion coefficient, $C_{\nu }$ is the specific heat capacity and *k* is the thermodynamic coefficient of isothermal compressibility. The propagation of an acoustic wave is defined by Newton’s second law [17,19]:

(3) $$\left(\nabla^{2}-\frac{1}{\nu_{s}^{2}}\frac{\partial^{2}}{\partial t^{2}}\right)\rho \left(\vec{r},t\right)=-\frac{\beta }{C_{p}}\frac{\partial^{2}}{\partial t^{2}}T\left(\vec{r},t\right),$$

where *T* represents the temperature rise (above its initial value). To solve (3), the Green’s function approach is commonly used to acquire a general forward solution which could be applied to the calculation of PA pressure induced by any arbitrary optical absorber [19]:

(4) $$\rho \left(\vec{r},t\right)=\frac{1}{4\pi \nu_{s}^{2}}\frac{\partial }{\partial t^{2}}\left[\frac{1}{\nu_{s}t}\int d{\vec{r}}^{\prime }\rho_{0}\left(\vec{r^{\prime }}\right)\delta \left(t-\frac{|\vec{r}-{\vec{r}}^{\prime }|}{\nu_{s}}\right)\right].$$

The high density of biological tissues results in the strong scattering of light, a major obstacle to PA and other optical imaging techniques, leading to a lack of focus at depth. Recent studies suggest the development of non-linear PA guided wave-front shaping (PAWS) to improve optical focusing [19,20]. Dual-pulse excitation based on the Grüneisen relaxation effect is then used with non-linear PA signals feedback for optimization. A clear optical focus of 5–7 μm (10 times better than the acoustic focus) and over 6000 times higher peak fluence is observed [20].

### 2.2. Excitation Sources

Pulsed lasers are frequently used as excitation sources in medical imaging. Optical to acoustic conversion efficiency in PA imaging is very low (<10${}^{-4}$ for liquids) [21], requiring high power sources. Pulsed lasers with a low duty cycle produce high peak output values with relatively low average power. This provides the advantages of strong photo-acoustic waves in biological tissues with a low heating effect (for repetition rates typically used in conventional PAI systems [22]). Pulsed lasers generate a high frequency PA signal that is easy to separate from low frequency environmental noise. This avoids the complex dependence upon thermal diffusion and the chopping frequency (or boundary condition) for the case of continuous excitation sources.

Careful consideration should be given to selecting an appropriate source for the right application, as the most expensive component in a PAI system is the pulsed laser. Taking into account non-irradiate relaxation time of photo-thermal de-excitation and stress confinement in biological tissues, nano-second pulse duration lasers are most suitable for PAI. Wavelength selection is determined by the optical absorption coefficients of the studied subjects, considering the optical absorption and scattering coefficients of the objects-located matrix. Selected wavelengths are typically in the visible or near-infrared spectral regions [9]. Laser energy per pulse should be in the order of mJ to sub-Joule for computed tomography (PACT or PAT), and in the order of μ J to sub-mJ for microscopy (PAM), endoscopy (PAE), and photo-acoustic flowmetry (PAF). The spatial distribution of the beam (TM00 mode) is important, especially in relation to optical resolution for PAM. To achieve high resolution and power utilisation efficiency in real-time imaging, the pulse repetition rate should be in the order of kHz or higher. Solid state lasers such as Q-switched Nd:YAG lasers are typically used in PAI, as they have a high power output for several wavelengths ranging from the ultraviolet (UV) to the near-infrared NIR [23]. Pulsed optical parametric oscillator (OPO) and dye lasers can continuously tune output wavelengths over a given range and are most suitable for multi-spectral PA imaging [24]. High-power pulsed laser diodes or diode modules have been paid special attention in PA measurements in the past twenty years for being low cost, compact, and most suitable for building portable PA microscopes [25]. Due to compactness, cost efficiency, high pulse frequency, and stability, fiber lasers have become popular for real-time, dynamic functional, and/or high-sensitivity molecular PAI [26].

### 2.3. Scanning PA Imaging Modalities

Scanning systems strongly affect imaging speed. Commonly used PAI scanning modalities include mechanical, optical and digital micro-mirror device (DMD) scanning [27]. Mechanical scanning has the advantage of a large scanning range and is traditionally used in PACT for animal whole-body and human part-body imaging. It is also used in PAE for acoustic and optical reflective mirror rotation within the probe as well as in PAM. Recently, galvanometers and micro-electro-mechanical-system (MEMS) mirrors have been used for PAI due to faster scanning rates and suitability for real-time imaging in PAM. DMDs, a widely used spatial light modulator, provides flexible and high-speed control of optical patterns that are delivered to the target. DMDs can be used for random-access light beam scanning in PAM and can deliver a frame rate more than ten times higher than raster scanning within arbitrarily shaped regions of interest within a 40 × 40 μm${}^{2}$ imaging area [28].

### 2.4. Acoustic Signal Detection

Piezoelectric ultrasonic transducers are most commonly used for PA detection because of their high sensitivity and simplicity [29]. Numerous studies utilized either a single element focused transducer or an array of flat/divergent transducers in a certain geometry (line, plane, cylindrical, spherical, Fresnel Zone Plate etc.) [13,30,31,32]. Song, et al. proposed a novel liquid acoustic lens that could minimize high acoustic impedance and low transmission efficiency issues in conventional solid-based transducers while providing a tunable focal length [33,34].

However, piezo-electric transducers have the drawbacks of their inherent resonant nature and a sensitivity that is proportional to their size. For applications with limited space, such as PAE, optical sensors provide an interesting alternative. One such option is a Michelson interferometer by Speirs et al. for pressure sensing, where the acoustic signal is extracted from the phase difference between the detection and reference beam [35].

Optical detectors have many advantages over piezoelectric transducers including good temporal resolution, wide bandwidth, small element size, and high stability to electrical/thermal artefacts, etc. [36,37]. An acoustic line detector using Mach-Zehnder interferometer developed by Paltauf et al. places the sensing arm much closer to the sample than the reference arm, altering the detected signal as PA pressure waves alter the relative path length [37].

Nuster et al. compared the Mach-Zehnder interferometer (MZI) with the Fabry-Perot interferometer (FPI) configuration by employing the two techniques simultaneously [37]. The acoustic signal was retrieved by using the FPI to demodulate the temporal derivative of the pressure-induced phase-change on the signal beam. A similar image quality was reported for the two devices, while the signal-to-noise ratio (SNR) of the FPI was lower. Furthermore, the signal beam of the MZI needs to be kept stationary for stability (not ideal for scanning large samples), while the FPI permits a scanning probe beam with the added advantages of low sensitivity to background noise and an easily transferrable setup [38].

**Figure 1:**
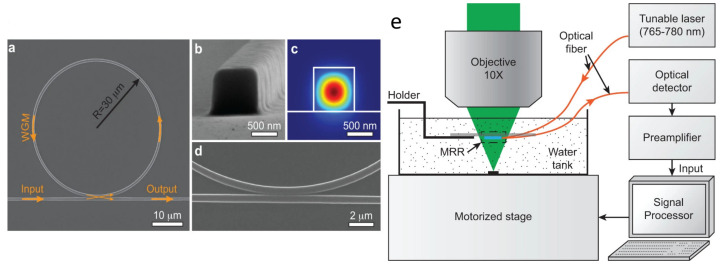
(**a**) Scanning electron micrograph of the MRR. (**b**) High-magnification view showing the square-shaped cross-section of the waveguide with a side length of 800 nm. (**c**) Calculated electric field distribution of the TM mode when the waveguide is immersed in water. (**d**) Close-up view of the gap between the ring and bus waveguides. (**e**) Experimental setup. Under CC BY-NC-ND 3.0 license from [16].

Micro-ring resonators (MRR) are another type of optical ultrasound (US) detector possessing the advantages of small dimensions, wide detection bandwidths, compatibility with high numerical aperture (NA) lenses, large fields of view (FOV), and ease of fabrication [39,40]. Consisting of a bus waveguide (serving as the input/output) and a coupled ring waveguide, an MRR recovers the acoustic signal from the optical intensity changes of the bus waveguide due to the acoustically-induced optical resonance in the coupled ring [39]. Hsieh et al. integrated the MRR using a dichroic filter, which transmits laser pulses at a single wavelength but performs as an ultrasonic source at another to achieve dual-mode PA/US imaging [41]. Li et al. constructed a confocal PAM system using an MRR with a bandwidth of 140 MHz. A saturation limit of 287 cm${}^{-1}$ and a lateral resolution of 2μm was obtained by the system shown in Figure 1 [16].

PA detection has also been achieved with polymer etalons [42], Fabry-Perot cavities [43] and Bragg gratings [44,45]. As another non-standard option, Taylor et al. developed an inherently efficient and low noise quantum memory based technique that is able to absorb selectively ultrasound tagged photons in a pair of atomic frequency combs, and recover them as a time-delayed photon echo [46]. In this manner, a recording of ultrasound-modulated sideband-to-carrier discrimination (49 dB) in highly scattering media has been demonstrated.

### 2.5. PA Image Contrast

Objects of interest in label-free PA imaging within biomedicine and life sciences are substances possessing different optical contrast values, associated with the optical absorption of chromophore’s content relative to the surrounding bio-tissue (or bio-matrix). The contrast substances within organisms are endogenous (or intrinsic) contrast agents, whereas exogenous contrast agents are usually non-organic substances added from outside. Contrast agents are key in determining the optimal modality and imaging quality for a given scenario.

**Figure 2:**
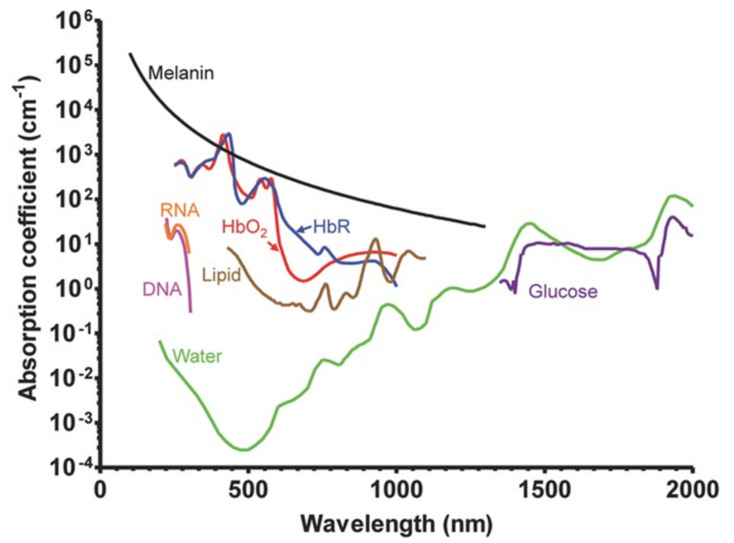
Absorption spectra of major endogenous contrast agents (chromophores) in biological tissue. Oxyhemoglobin, red line (150 g/L in blood); Deoxy-hemoglobin, blue line (150 g/L in blood); Lipid, brown line (20% by volume in tissue); Water, green line (80% by volume in tissue); DNA, magenta line (1 g/L in cell nuclei); RNA, orange line (1 g/L in cell nuclei); Melanin, black line (14.3 g/L in medium human skin); Glucose, purple line (720 mg/L in blood). Reprinted from [47], Copyright (2013), with permission from John Wiley and Sons.

The absorption of endogenous contrast agents in bio-tissues depends upon the excitation wavelengths (Figure 2). In the UV region these agents include Melanin, DNA/RNA, cytochrome c, myoglobin, and haemoglobin. These also possess strong optical absorption and serve as contrast agents from visible to short NIR wavelengths (<1200 nm), including lipids but excluding DNA/RNA. For longer wavelengths, water and glucose are good contrast agents. It is important to mention that the oxygenation of haemoglobin impacts upon optical absorption in visible wavelengths. For this reason it is an important contrast agent in functional and metabolic imaging, as well as distinguishing malignant tumours (or cancers) from those that are benign. Endogenous contrast agents are fundamental for label-free imaging, preferred in biomedical research for the lack of disturbing cells or micro-environments in the original bio-tissue and aren’t time-consuming when seeking regulatory approval.

Exogenous contrast agents have important PA applications as they absorb optical radiation more strongly than endogenous agents, greatly increasing imaging depth in the body. In addition, in some spectral regions the absorption peak of some exogenous contrast agents (e.g., nano-particles) can be continuously tuned to enable imaging at desired wavelengths. Endogenous agents can be also conjugated with targeting molecules (e.g., antibodies) to selectively bind to specific cells, greatly improving imaging contrast. Besides PAI, exogenous contrast agents can be used in multiple imaging modalities (optical, florescence, etc.), to carry drugs to specific cells, and guiding or monitoring thermos-therapeutic procedures. To date, many exogenous contrast agents have been developed in PAI [48], which can be roughly categorised as organic dyes, nano-particles, fluorescent proteins and reporter gene products [47].

## 3. Current Photoacoustic Imaging Modalities

### 3.1. PA Computed Tomography (PACT/PAT)

**Figure 3:**
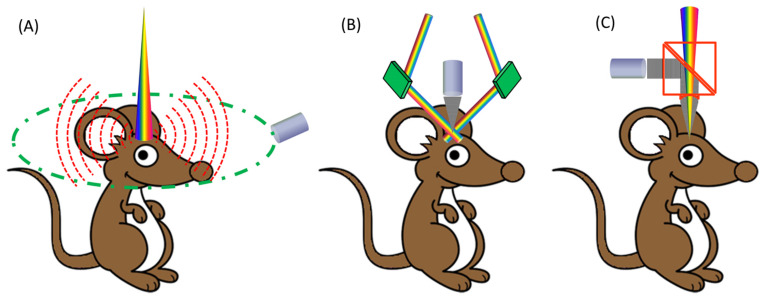
Materialization of label-free cerebral vascular imaging [50]. (**A**) Schematic of PACT: a pulsed laser beam from a wavelength-tunable laser system is expanded and homogenized to provide uniform illumination covering the entire region of interest (ROI). A circular-scanning single-element ultrasonic transducer [51] or a stationary transducer array [52] is placed around the tissue to receive PA waves. (**B**) Schematic of dark-field acoustic-resolution microscopy (AR-PAM): a pulsed laser beam is reshaped by a conical lens to form a ring pattern and then is weakly focused beneath the tissue to overlap the tight ultrasonic focus. (**C**) Schematic of optical-resolution microscopy (OR-PAM): a diffraction-limited bright-field optical illumination, ten times smaller in diameter than the acoustic focus, is adopted to achieve optical resolution. Adapted under CC BY 4.0 license from [50].

PACT is similar to X-ray computed tomography, usually employing an ultrasonic transducer or array to accept PA waves from a studied object at multiple view angles before reconstructing the distribution of optical absorption (Figure 3). The simplest case would be shining a laser beam onto a layered medium while detecting the acoustic signal corresponding to energy absorption, which can be regarded as One-Dimensional (1D) Tomography. The different structure and absorption information at different depths can be extracted from the acquired temporal PA wave, hence the name of “depth profiling” [49]. Tomographic imaging employs multiple transducers or arrays to deliver 2D or 3D images.

In early stage experiments, PAT systems were set up using a single element transducer and tomographic imaging was accomplished by scanning the transducer using translational or rotational stages [51,53].

**Figure 4:**
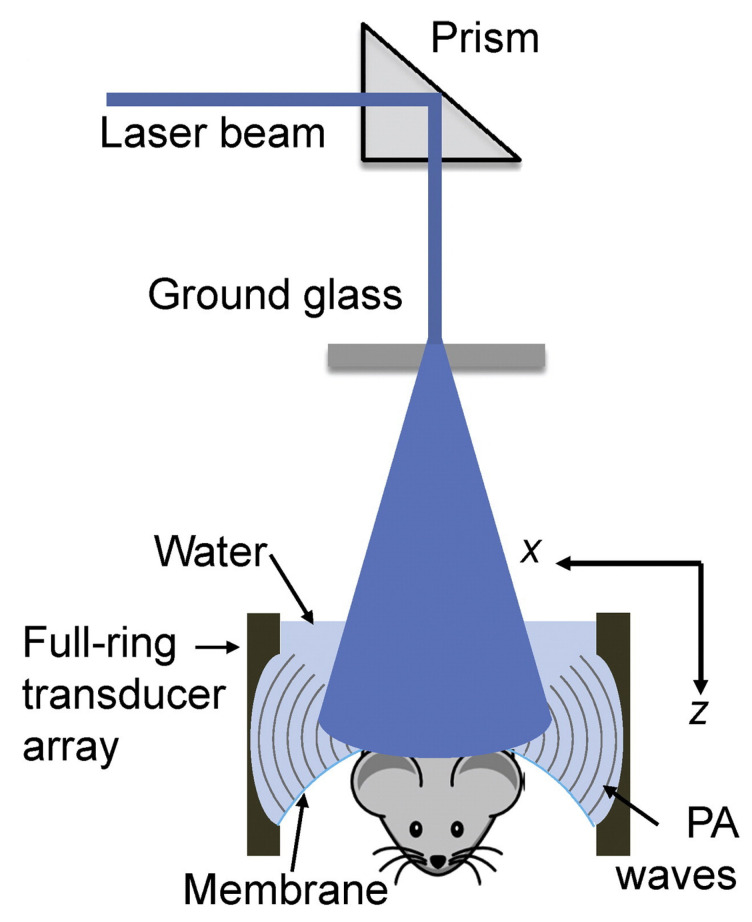
Schematic of the PACT setup. An OPO laser pumped by a Nd:YAG laser with third harmonic generation (355 nm) provides pulses with tunable wavelengths (420–680 nm), with a pulse duration of 6 ns and repetition frequency of 10 Hz. PA signals were detected by a 5 cm diameter full-ring ultrasonic transducer array with 512 elements. X and Z represents axis of the experimental setup. Reprinted from [54], Copyright (2013), with permission from Elsevier.

As technology developed, more sophisticated arrays and/or multiple illumination directions were applied for deeper penetration, higher resolution, faster imaging speed and higher frame-rate. Figure 4 shows a circular/cylindrical shaped transducer array that could be used for finer acoustic focus and resolution. A 5 cm-diameter circular transducer array with 512 elements was employed by Yao et al. with a reported lateral resolution of 0.25 mm and an axial resolution of 0.1 mm within the focal zone [54,55].

**Figure 5:**
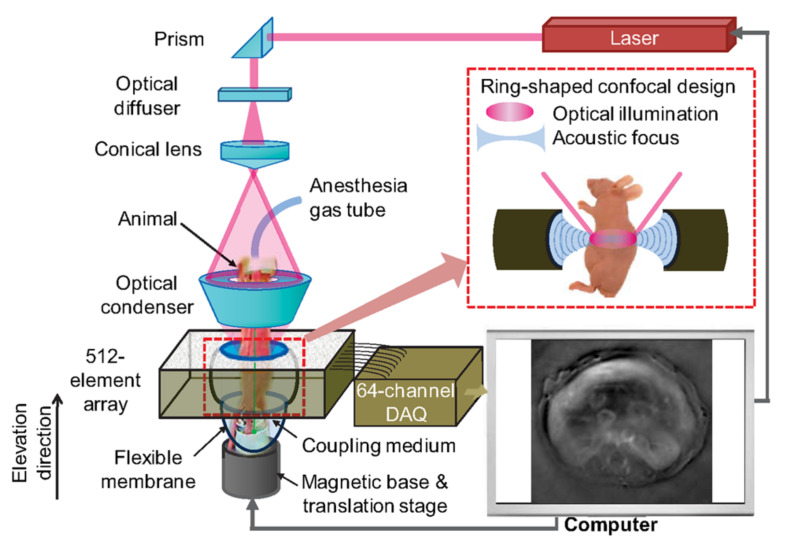
Full-ring confocal whole-body photoacoustic computed tomography (RC-PACT) system schematic [56]. A Ti-Sapphire laser with 12 ns pulse duration and 10 Hz pulse repetition frequency was used as the irradiation source. The laser beam was first homogenized using an optical diffuser, then passed through a conical lens to form ring-shaped light. This was then focused using an optical condenser made from acrylic to project a light band around the animal. Reprinted, with permission, from [56].

Figure 5 Wang et al. illustrates a ring-shaped confocal (RC) PACT small-animal whole-body imaging system [56], based on a confocal design of free-space ring-shaped light illumination and a 512-element full-ring ultrasonic array for signal detection. The free-space light illumination maximizes light delivery efficiency and the full-ring signal detection ensures a full two-dimensional view aperture for accurate image reconstruction. Using cylindrically focused array elements, it can image a thin cross section with 0.10 to 0.25 mm in-plane resolution and 1.6 s/frame acquisition time. By translating the mouse in the direction of elevation, RC-PACT provides a series of cross-sectional images of the brain, liver, kidneys, and bladder.

**Figure 6:**
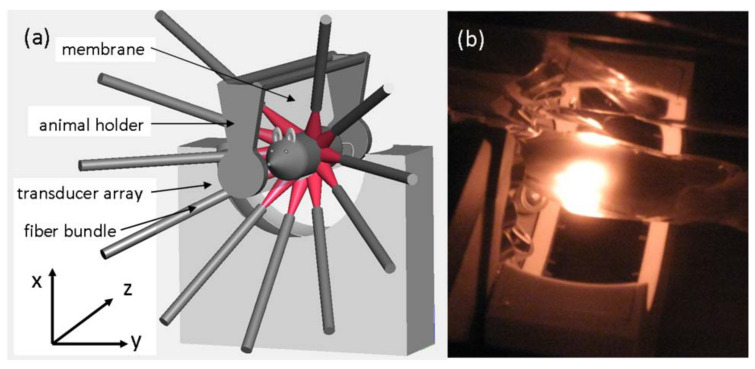
(**a**) Schematic of an optical fiber array to homogeneously illuminate animal and a curved array of wide-band and cylindrically focused transducer array enabling parallel data acquisition. (**b**) Small animal during a scan. Reprinted with permission from [57] © Optica Publishing Group.

Figure 6 shows a typical setup with several fibre bundle arms evenly spread around the sample for more uniform light distribution. A custom-made 64-element ultrasound transducer (UST) array covered $172^{\circ }$ with a centre frequency of 5 MHz providing an effective in-plane resolution of 150μm [57]. A real-time handheld device integrating a laser fibre bundle with a 128-element spherical concave UST array was developed, the estimated axial resolution is around 115μm and a frame rate of 50 Hz is achieved [58]. A similar technique was used to image blood vessels within the human finger in vivo, with a focus on vascularity across the interphalangeal joints. The resultant approach can monitor vascular abnormalities such as those arising from angiogenesis or associated with inflammatory rheumatic disease [59].

**Figure 7:**
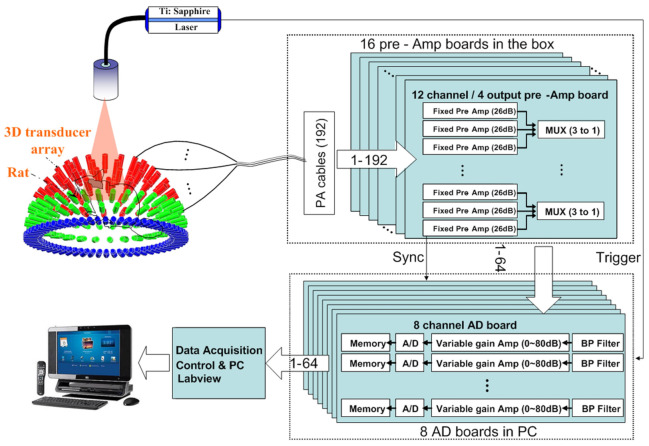
4D PAT system schematic. A tunable pulsed laser (Ti: Sapphire, 690–950 nm) was used as a light source for tissue surfaces. A 192 element 3D sphere transducer array was used to capture photo-acoustic signals generated by the laser light. Under CC BY-NC-ND 3.0 license from [60].

A more complex apparatus obtained using a spherical array of ultrasonic detectors is illustrated in Figure 7, which can form so called 4D PAT by integrating time resolutions with 3D spatial resolutions. The average time resolution is 330 ms (limited by the 10 Hz laser repetition and improvable by substituting a laser with a higher pulse repetition rate) and spatial resolutions of 0.19 mm in the *x*-*y* direction and 0.27 mm in the *z*-axis [60,61]. While transducer arrays with large numbers of elements provide quality PAT images, the system and acquired data complexity need to be reduced by using either an electronic or MEMS based acoustic multiplexer [62]. The 4D PAT technique can be used to generate motion pictures of imaged tissue, enabling the real time tracking of dynamic physiological and pathological processes at hundred micrometer-millisecond resolutions with an imaging depth in centimetres.

In 2014, Tang et al. developed a more miniature and portable PACT system with an observed frame rate of 3.33 frames/s and a stated potential of 10 frames/s [63]. This level of frame rate enables real-time functional imaging for vasculature, hemodynamic and metabolism monitoring, along with mammography for breast cancer [60,64,65].

PAT can be integrated with other techniques for multi-modality imaging. For example, the combination with a clinical ultrasound array provides complementary contrast to a standard pulse echo ultrasound. Kim et al. described a PA system with two laser fibre bundles attached to the side of a clinical linear ultrasonic transducer array probe of 4–8 MHz bandwidth [66]. An imaging depth of 5.2 cm in chicken breast at 650 nm was achieved with the potential of extending it to 7.4 cm for higher laser powers. The PA signals were averaged 100 times for better signal-to-noise ratios (SNR) however this limited the frame rate of imaging significantly [67]. Montilla et al. improved laser delivery using a PA enabling device (PED) based on an optically transparent acoustic reflector as an accessory for commercially available ultrasonic transducers. The PED eliminated the unwanted dark regions while shortening the optical path and reducing losses due to scattering. A signal enhancement of 18 dB at 5 mm depth and 6 dB at 20 mm depth was observed [68].

PA image reconstruction from data collected with transducer arrays has been extensively researched. Major approaches include delay and sum (DAS), universal back-projection, frequency-domain time reversal, model-based (iterative), compressed sensing algorithms etc. [66,69,70,71,72]. Treeby, et al. have developed a MATLAB toolbox called *k*-Wave for PA simulation and reconstruction [73].

### 3.2. MultiSpectral Photoacoustic Tomography (MSOT)

As biological tissues always contains multiple spectrally distinct chromophores, in general, more than two optical wavelengths are necessary to perform efficient spectral unmixing of the different absorbing substances. By performing multi-spectral scan over the imaging target, Multi-spectral opto-acoustic tomography (MSOT) is able to provide spectral absorption contrast for the visualization of biomedical information aided by a wide range of endogenous/exogenous contrast agents. MSOT is wide-ranging and useful in applications including cancer detection, high-resolution deep tissue imaging, drug delivery, and cardiovascular disease detection etc. [57,58,74,75,76,77,78,79,80].

A typical experimental setup [57,81] with excitation and detection schema are shown in Figure 6. MSOT enables operation in real-time mode by capturing single cross-sectional images of less than 1 ms from living small animals (e.g., mouse) and other tissues of similar dimensions. At the core of the method is the illumination of the object using multiple wavelengths in order to resolve spectrally distinct biomarkers over background tissue chromophores. The system allows the horizontal placement of a mouse in the imaging chamber and three-dimensional scanning of the entire body without the need to immerse the mouse in water. Overall, the entire protocol can be completed within 15–30 min for acquisition of a whole-body multispectral data set from a living mouse. Dean-Ben and Razansky demonstrated a hand-held multi-spectral scanner to obtain time-resolved volumetric blood oxygenation maps in deep human vessels and real-time tracking of contrast agent distribution in a murine model in vivo, which could be thought as 5D PAT as it integrated time and spectral information together with 3D spatial tomography [82]. Volumetric MSOT can provide real-time imaging and characterization of the entire carotid bifurcation area across three dimensions simultaneously captured in a single volumetric image frame [83]. This imaging technique has decreased motion-related artifacts compared to conventional clinical imaging methods, having the potential for noninvasive functional assessment of cardiovascular disease.

Table 1 shows the advantages of MSOT compared with existing methods of visualization.

### 3.3. Photoacoustic Microscopy (PAM)

**Figure 8:**
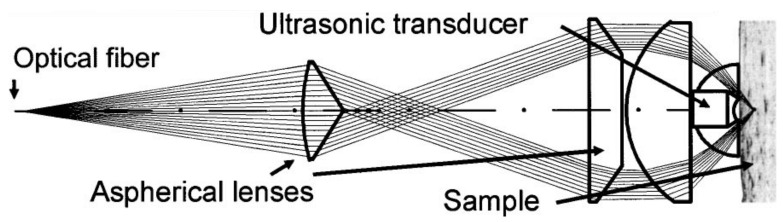
Early stage black-field PAM setup to image mouse ear vasculature. Laser light from the fiber was expanded by a conical lens and focused through an optical condenser. The optical focal region overlaps the focal spot of the ultrasonic transducer, thus forming a confocal optical dark-field illumination and ultrasonic detection configuration. Reprinted with permission from [85] © Optica Publishing Group.

Photoacoustic microscopy is another major field for PA imaging. While conventional imaging techniques such as confocal microscopy and optical coherent tomography do not sense purely optical absorption and cannot image object deeper than 1 mm inside tissues because of highly optical scattering, PA microscopy benefits from greater penetration depth, higher optical-absorption contrast and higher ultrasonic resolution. Early PAM systems (Figure 8) were reported to image blood micro-vessels easily in skin depths of over 3 mm with a lateral resolution of 45μm [85]. Nowadays, PAM achieves images with sub-micrometre resolution at around 1 mm tissue depth [11].

A number of different modalities have been developed in the last two decades, with a range of resolution and penetration scales including acoustic-resolution PAM (AR-PAM), optical-resolution PAM (OR-PAM), sub-wavelength PAM (SW-PAM), double-illumination PAM (DI-PAM), photo-imprint PAM (PI-PAM), variable focus PAM, Multiview OR-PAM, and Grüeneisen-Relaxation PAM et al. [31,47,86,87,88,89,90].

#### 3.3.1. Acoustic Resolution Photoacoustic Microscopy (AR-PAM)

The category of AR-PAM is based on the configuration of having a smaller focus region for the ultrasonic transducer than the pulsed laser beam [47].

**Figure 9:**
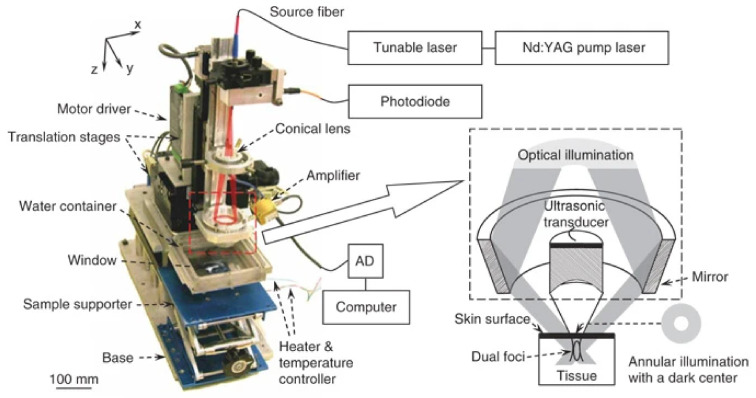
An AR-PAM system presented by Zhang et al. with ring-mirror [91]. The components within the dashed box were mechanically translated along an *x*-*y* plane with the bottom of the mirror and the ultrasonic transducer was immersed in water. A window at the bottom of the water container was sealed with an optically and ultrasonically transparent disposable polyethylene membrane (thickness, 0.044 mm). The sample was placed between the water container and the sample supporter for imaging covered with ultrasound gel. Reprinted by permission from Springer Nature: Nature Biotechnology [91] (Functional photoacoustic microscopy forhigh-resolution and noninvasive in vivoimaging, Hao F Zhang et al), COPYRIGHT (2006).

Zhang et al. presented a functional microscope (Figure 9) in 2006 [91]. Dark-field imaging (detection of reflection rather than transmission) was used for better imaging quality. As the ultrasonic transducer is at a better focus than the optical pulse, the resolution of the system is dominated by features of the transducer: a wider bandwidth and a larger numerical aperture would help to improve the resolution. The wider the bandwidth is, the higher the centre frequency will be for the ultrasonic transducer and hence more severe attenuation ultrasonic signals will suffer from. A lateral resolution of 15 μm and spatial resolution of 45 μm in the focal zone was reported with imaging depths as high as 3 mm. Melanoma and haemoglobin are the local contrast agents with very strong absorption at various yet distinct wavelengths [91,92].

However, the application of previous systems for functional imaging is limited due to the lack of scanning speed. It would take up to 20 min for a full scan of an area around 8 × 8 mm${}^{2}$. The main obstacles are laser repetition rate and mechanical scanning speed, as a single A-scan would only take about 2 μs to image up to 3 mm deep (speed of sound at around 1.5 mm/μs in water/tissue) [91,92].

Simply by utilizing a faster scanning stage-voice coil stage along with a laser with repetition rates up to 30 kHz instead of 10 Hz, a new PA microscopy system could achieve 40 frames of B-scan per second [93].

#### 3.3.2. Optical Resolution Photoacoustic Microscopy (OR-PAM)

While AR-PAM provides a reasonable spatial resolution at organ and vascular scales, to further enhance the imaging quality for cellular or sub-cellular structures would actually require the central frequency of the ultrasonic transducer to exceed 300 MHz at which the attenuation is too severe for the acoustic wave to propagate at all [31]. The first OR-PAM system was developed to solve this problem, demonstrating a lateral resolution of 5μm and an imaging depth of 0.2 mm to 0.7 mm. The enhancement of resolution was the result of a better focused laser beam. The optical objective lens and ultrasonic transducer are coaxially and confocally aligned for optimum imaging acquisition [94].

**Figure 10:**
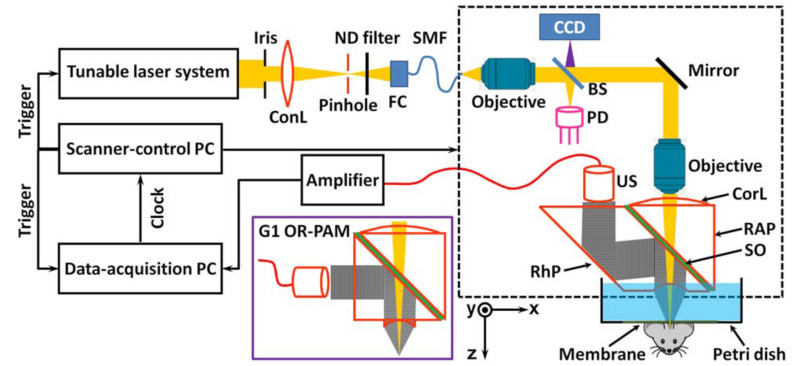
Second Generation OR-PAM with rhomboid prism for better acoustic reception [95]. US—ultrasonic transducer; ConL—condenser lens; ND—neutral density; FC—fiber collimator; SM—single-mode fiber; CCD—charge-coupled device; BS—beam splitter; PD—photodiode; CorL—correction lens; RAP—right-angle prism; SO—silicone oil; RhP—rhomboid prism. Reprinted with permission from [95] © Optica Publishing Group.

The second generation of the OR-PAM system (Figure 10) replaced the right-angle prism in contact with the acoustic transducer with a rhomboid shaped prism. The second reflection of the rhomboid prism transformed most of the energy lost in the shear wave from first reflection back into the longitude acoustic signal and therefore improved the sensitivity of detection. As deep as 1.2 mm imaging ability was experimentally proved with lateral resolution of 2.65μm [95].

**Figure 11:**
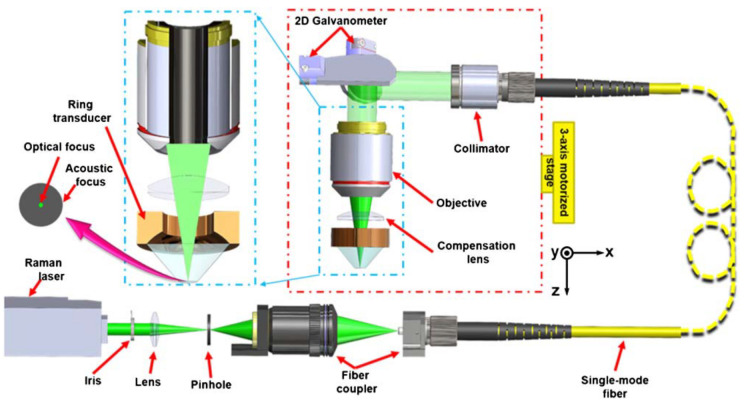
Scanning OR-PAM with a 2D Galvanometer for enhanced imaging speed [97], employing a ring-shaped focused ultrasonic transducer for reflection-mode ultrasound detection. Reprinted with permission from [97] © Optica Publishing Group.

Since the second generation of OR-PAM, the use of a 2D scanning galvanometer (Figure 11), enabled both bright-field and dark-field modalities to be 20 times faster, while the lateral resolution was enhanced to 500 nm [96,97]. Scanning has also been accomplished by a 2D high-speed MEMS scanner [98].

A number of other PAM techniques have been developed in the last decade. Hai et al. developed near-infrared (NIR) OR-PAM with a lateral resolution of 6.2μm (less than visible region OR-PAM) and an imaging depth of 3.2 mm due to lower optical decay in the NIR region [99]. A PI-PAM technique by Yao et al. could improve resolution significantly down to 200 nm using laser double-excitation to partially and non-homogeneously bleach molecules in the illumination zone so that molecule absorption at centre upon second excitation are lessened to produce a signal difference between two output pulses [87]. Jiang et al. integrated AR-PAM and OR-PAM for a multiscale PAM with a tunable lateral resolution between 1μm to 44.8μm through a variable focus lens [88,100,101]. A quantitative microscopic method namely PACM was developed by Yao et al. employing a model-based inverse reconstruction algorithm to quantify the optical absorption of targets [102]. To improve the working distance and render it more convenient for the application at hand, Wang et al. [103] developed a reflection-mode optical-resolution PA microscopy based on a reflective objective, with a working distance as large as 6 mm. It provided a lateral resolution of 1.2μm and a penetration depth of 0.9 mm in biological tissues at 580 nm.

Axial resolution in the above PAM modalities depends upon pulse duration, commonly worse than 15μm in biological tissues and generated by nanosecond pulses. To break through this limit and achieve resolutions approaching sub-cellular levels, Wang et al. developed Grueneisen Relaxation Photoacoustic Microscopy [90] which reached axial resolutions of 2.3μm and simultaneously reached lateral resolutions between 0.63μm and 0.41 μm. In addition, the optical sectioning capability facilitates the measurement of the absolute absorption coefficient without fluence calibration. Shelton and Mattison et al. developed a transient absorption ultrasonic microscopy (TAUM) that combines PAM with pump-probe spectroscopy to encode a PA signal at overlap of a pump beam and a probe beam [104,105], delivering 1.5μm axial resolution [106]. Zhu et al. [89] developed multi-view optical resolution PA microscopy to experimentally demonstrate an isotropic optical resolution of 4.6μm in three dimensions. Strohm et al. directly used ultra-high frequency (UHF) wide-bandwidth transducers to receive PA signals and reconstruct images [107], the achieved lateral and axial resolutions approached 1 μm but had reduced penetration depth.

A most novel study considering nano-scale resolution (PA nano-scopy) [108] is already challenging PAM. This is based on PA non-linearity, where multiple signals are successively excited with increasing pulse energy at each scanning position. Because of optical saturation or nonlinear thermal expansion, the PA amplitude depends on the non-linear incident optical fluence. The high-order dependence, quantified by polynomial fitting, provides 88 nm super-resolution imaging with optical sectioning.

### 3.4. Photoacoustic Endoscopy (PAE)

**Figure 12:**
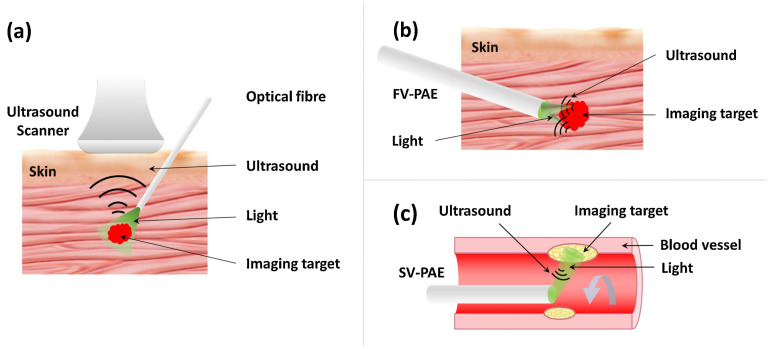
Major embodiments of minimally invasive photoacoustic imaging techniques: (**a**) Interventional photoacoustic imaging (iPAI), (**b**) Forward-viewing photoacoustic endoscopy (FV-PAE), and (**c**) Side-viewing photoacoustic endoscopy (SV-PAE). Reprinted under CC BY 4.0 license from [109].

Photoacoustic endoscopy is a variation of PAT uniquely designed for imaging internal organs, in which the detector and source (or delivery mechanism) are contained within a miniature probe (Figure 12) that can be inserted into the body [109]. This is a relatively novel application of the PA effect [110,111,112] and two common designs can be seen in Ref. [113]. Yang et al. developed a system delivering high-repetition-rate laser pulses to the probe (diameters ranging from 2.5 mm to 12.7 mm) through a single multimode optical fibre [111]. A ring-shaped ultrasonic transducer was placed coaxially, holding the fibre for optimized detection sensitivity. Acoustically- and optically-reflective mirrors were employed for illumination and signal detection. Driven by a rotational motor and a linear motor, the PAE probe can scan both cross-sectional images and volumetric images. A dual-mode PA and US endoscopic probe was developed to better identify and characterize the anatomical structures of the esophageal lumen. Blood vessels with diameters as small as 190μm are able to be resolved using this approach [114].

The typical B-scan frame rate is circa 5 Hz, imaging depth is circa 7 mm and spatial resolution in the order of 100μm or smaller.

**Figure 13:**
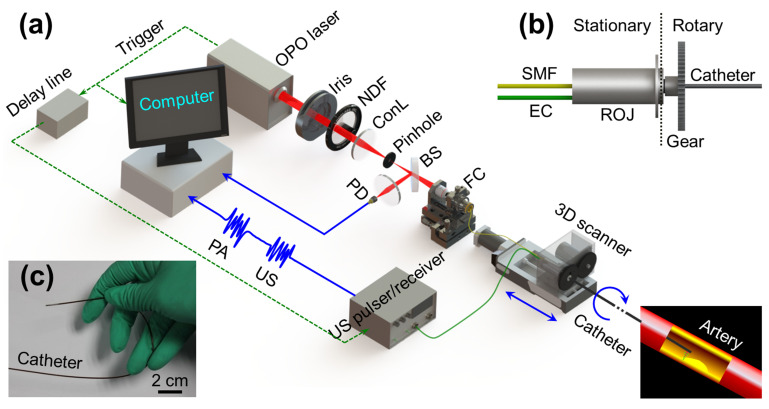
Experimental setup of OR-PAT with catheter for intravascular imaging [115]. (**a**) Overall architecture of the system. (**b**) Schematic of the rotational mechanism of the catheter. (**c**) A demonstration of catheter flexibility. OPO—optical parametric oscillator, NDF—neutral density filter, ConL—condenser lens, BS—beam splitter, PD—photodiode, FC—fiber coupler, US—ultrasonics, PA—photoacoustics, SMF—single mode fiber, EC—electrical cable, 3D scanner, consisting of an optical-electric rotary joint (ROJ), a step motor, and a motorized pull-back stage. Reprinted under CC BY 4.0 license from [115].

Similar to endoscopic devices, intravascular PA systems also require the use of catheters. Bai et al. presented an optical resolution photo-acoustic tomography system (OR-PAT) for intravascular imaging (Figure 13) offering lateral resolutions as low as 19.6μm [115]. Jansen et al. developed intravascular photo-acoustic (IVPA) imaging of human coronary atherosclerotic plaques. A 1.25 mm diameter intravascular imaging catheter was built, comprising an angle-polished optical fiber adjacent to a 30 MHz ultrasound transducer. Specific photo-acoustic imaging of lipid content, a key factor in vulnerable plaques that may lead to myocardial infarction, was achieved by spectroscopic imaging at wavelengths between 1180 and 1230 [116]. However, IVPA systems suffered from slow imaging speed (50 s/frame) due to the lack of a suitable source for the high-speed excitation of molecular overtone vibrations. Recently, Wang et al. [117] designed and developed of a kHz repetition rate Raman laser to improve intravascular photoacoustic imaging speed to 1.0 frame/s, narrowing the gap in translating IVPA to a clinical setting. Li et al. [118] developed a 0.9 mm catheter for IVPA imaging, smaller than the critical clinical translation requirement of 1 mm. They employed an ns-pulsed OPO laser with a 1-kHz pulse repetition rate to achieve high speed imaging, which can be further translated to in vivo applications.

### 3.5. Photoacoustic Flow Cytometry (PAFC)

**Figure 14:**
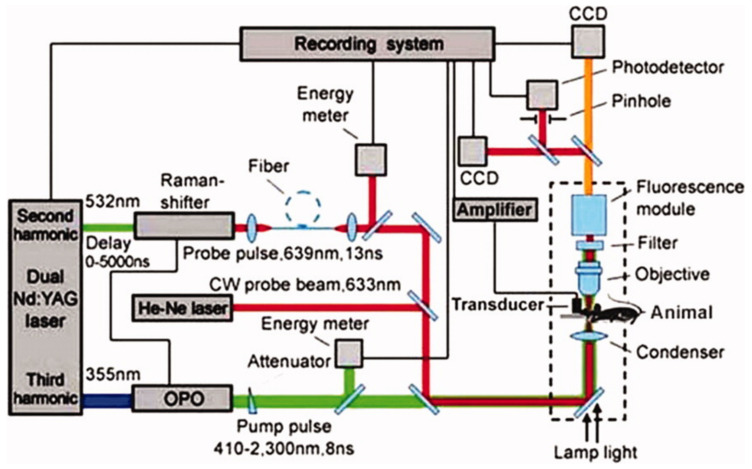
Schematic of an in vivo flow cytometer integrating PA, PT, fluorescence, and transmission digital microscope (TDM) detection techniques [122]. The cytometer was built on the technical platform of an upright Olympus BX51 microscope with incorporated PA, PT, fluorescent, and TDM modules. Reprinted from [122], Copyright (2008), with permission from John Wiley and Sons.

Although flow cytometry is not a traditional imaging technique, it can monitor and count biological cells in a circulating system such as blood or lymph vessels to diagnose diseases in their early stages. Different to conventional flow cytometry sensing, optical scattering or fluorescence, PAFC detects changes in absorption within circulating vessels. This is possible due to intrinsic cells [119] such as red blood cells, white cells (with negative contrast), tumor cells, melanoma cells (with positive contrast), or cells to be bio-conjugated with exogenous contrast agents [120,121]. Hence, PAFC has higher sensitivity and specificity for cell detection. PAFC uses a nanosecond pulsed laser with pulse energy in the order of micro-joules and 10 kHz or more pulse repetition rate to exit selected vessels. The cell generated time-solved PA signals are detected by a focused ultrasonic transducer (with a central frequency from a few MHz to tens of MHz) attached to the skin. PAFC has been used to investigate cells in blood vessels with diameters between 30 and 1000μm in skin depth from sub-mm to 4 mm. The spatial resolution of PAFC is dependant upon optical focusing in superficial vessels and acoustic focusing in deeper vessels. PAFC is easily integrated with optical microscopy (Figure 14) that delivers a more powerful tool capable of studying PA, photothermal, fluorescent and optical scattering properties of cells simultaneously [122]. Beyond this concise summary, an excellent review by Galanzha and Zharov is recommended to the reader for consideration [123].

Table 2 summarizes the main features of PAI techniques. PACT is highly sensitive to the rich optical absorption contrast of biological tissues and is inherently well suited for functional, molecular, metabolic and histological imaging (through endogenous contrast) and for molecular and neuronal imaging (through exogenous contrast).

MSOT additionally provides real-time functional information and the evaluation of physiologic conditions with label-free mapping of blood oxygen saturation in tissues [83].

PAM, in contrast to PACT, detects signals generated from the transducer focus and thus does not require inverse reconstruction algorithms [124]. PAM takes advantage of weak acoustic scattering in tissue, thus piercing the optical diffusion limit to provide high-resolution images at the imaging depths of up to a few millimeters.

PAFC provided the opportunity for the dynamic study of blood rheology including red blood cell aggregation and clot formation in different medical conditions [123].

The major advantage of PAE is the penetration depth, which has great potential for early-stage tumor detection, the accurate diagnosis of submucosal lesions, gastrointestinal and cardiovascular imaging.

## 4. Biomedical Applications

### 4.1. Brain Imaging

In vivo structural and functional brain imaging is one of the most important biomedical applications of PA imaging. Extensive research and reviews have been published in this field [51,54,63,65,75,100,102,125,126,127]. Wang et al. reported an early-stage PAT system in structural and functional imaging of mouse brain vasculature (blood flow rate being related to neural activities). However, due to mechanical limitations, it took 16 min to acquire each image at a spatial resolution of 0.2 mm [51]. A more recent setup by Nasiriavanaki et al. employed PAT with a 512-element ring transducer array at an in-plane spatial resolution of 100μm to perform functional analysis of resting-state functional connectivity (RSFC) on mice. RSFC is of great importance and high potential in the study of strokes, Alzheimer’s disease, multiple sclerosis, autism, epilepsy, etc. Compared with other functional imaging techniques, functional PAT (fcPAT) provides higher resolution and molecular imaging capability when combined with bio-markers [65]. Despite the limitation of relatively low resolution, non-uniform light illumination and fixation difficulties, Tang et al. described a more real-time miniature cap-like wearable PAT system with a 64-element ring UST array. Imaging rates of 10 Hz on a 15 mm × 30 mm area were achieved for monitoring hemodynamics in rat brains [63]. PAM has excellent potential for stroke monitoring in small animals at different stages [128]. Unfortunately, there are still limitations to photo-acoustic studies of the human brain due to the challenge posed by the skull.

**Figure 15:**
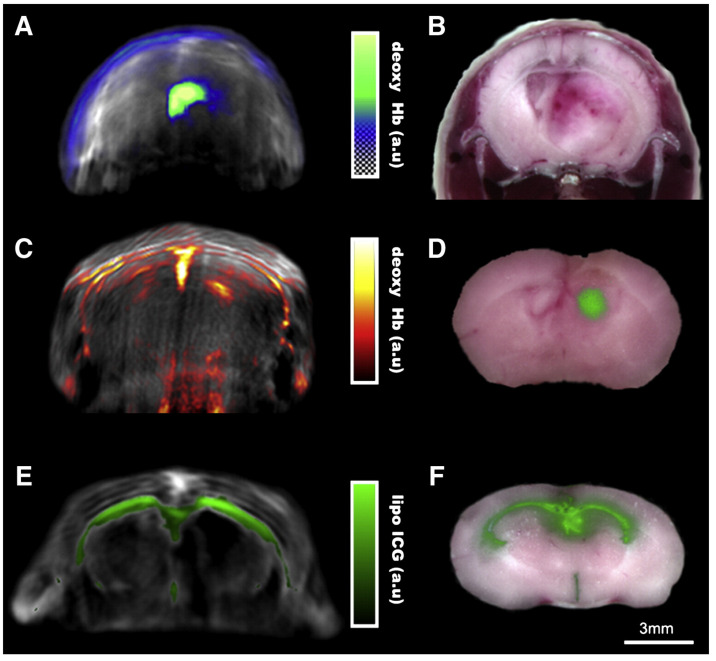
Multispectral unmixing of glioblastoma and liposomal ICG distribution in brain. Panel (**A**) shows the spectrally unmixed deoxy-hemoglobin pseudocolor overlay on an 800 nm single wavelength MSOT image from an animal 34 days following implantation with U87 glioblastoma cells with a corresponding ex vivo cryosection in (**B**). Panel (**C**) shows a deoxy-hemoglobin image 16 days following implantation following a 10% carbon dioxide challenge with corresponding ex vivo fluorescence image from IntegriSense750 showing tumor size and location (panel (**D**)). Panels (**E**,**F**) show a mouse injected intraventricularly with ICG encapsulated into liposomes. Panel (**E**) shows 900 nm single wavelength MSOT image (grayscale) with an overlay (green) of the spectrally-resolved liposome-ICG signal. Panel (**F**) shows the equivalent cryoslice with an overlay of the fluorescence from the injected particles. Reprinted from [75], Copyright (2013), with permission from Elsevier.

With the ability of multi-spectral analysis, MSOT has plenty of applications within brain imaging. Burton et al. tested an MSOT system with a tuneable laser and a 64-element cylindrical UST array at 5 MHz central frequency to detect glioblastoma, aided by injected Indocyanine green (ICG) as contrast agent. The spatial resolution was circa 150μm, determined by the 5 MHz centre frequency and could be improved to 50μm if 30 MHz was used instead. The reconstructed MSOT image (Figure 15) suggested a strong correlation between the location of tumour cells and deoxygenated haemoglobin concentration [75].

Though haemoglobin and chemical dyes are the main contrast agents available for brain imaging, other techniques were developed. Most recently Sela et al. used functional opto-acoustic neuro-tomography (FONT) to monitor calcium dynamics during neuro-activities through genetically coded calcium indicators. Real-time monitoring at frame-rate of 100 Hz over 200 m${}^{3}$ volumes was claimed along with very high axial and lateral resolutions of 52μm and 71μm, respectively [127].

In contrast to brain imaging for small animals, photo-acoustically imaging the adult human brain is much more difficult due to the greater thickness of the human skull. This thickness is (7–11 mm, compared to less than 1 mm for small animals such as mice and rats) [129]. The human skull strongly attenuates incident light and emitted PA waves, resulting in 2.1% light transmittance at 1064 nm [130] and 20 dB/cm acoustic attenuation at 1 MHz [131]. Despite the viability of the photo-acoustic effect within brain tissue, ultrasonic propagation through the skull is challenging due to the acoustic impedance disparity with surrounding tissues [128]. Nie et al. developed a photon recycler to increase light transmittance through skull which improve the PA SNR by a factor of 2.4 [130], making it possible to study functional activity within the human cerebral cortex. On the other hand, as the skull thickness of neonatal infant is much thinner than that of adult (only about 1.3 mm [132]), PA imaging can be used to monitor neonatal brain [132,133]. Wang et al. [132] used PAT to image blood vessels through infant skull with a 50μm axial resolution and 420μm lateral resolution. The imaging depth can be as deep as 21 mm or more in the case of NIR PA excitation. PAT has also been used to image the brain cortex of larger animals (such as monkeys) through the intact scalp and skull ex vivo [134], which is a good simulation for the case of studying the brain of human children.

### 4.2. Vascular Imaging

Cardiovascular disease has been listed as the number one cause of death by the World Health Organization (WHO) for the past decade. Lipid-rich atherosclerosis plaques are found to be causing cardiovascular disease [13] and PA techniques are very suitable for imaging lipids due to their high optical absorption at 1150–1250 nm and 1.7μm with respect to the tissue background (which is mostly water) [4,118,135,136,137]. Traditionally angiograms were applied, however they failed to differentiate the culprit lesion from stenosis-led outcomes [138]. More modern standard intravascular evaluations have been performed by intravascular ultrasound (IVUS) with 100μm resolution and 7 mm penetration depth, however, the sensitivity and specificity of plaque content are limited [139]. Other intravascular optical imaging techniques such as intravascular optical coherence tomography (IVOCT) and near-infrared reflection (IVNIR) have imaging depth limitations (see Table 3) like their common versions [140,141].

**Figure 16:**
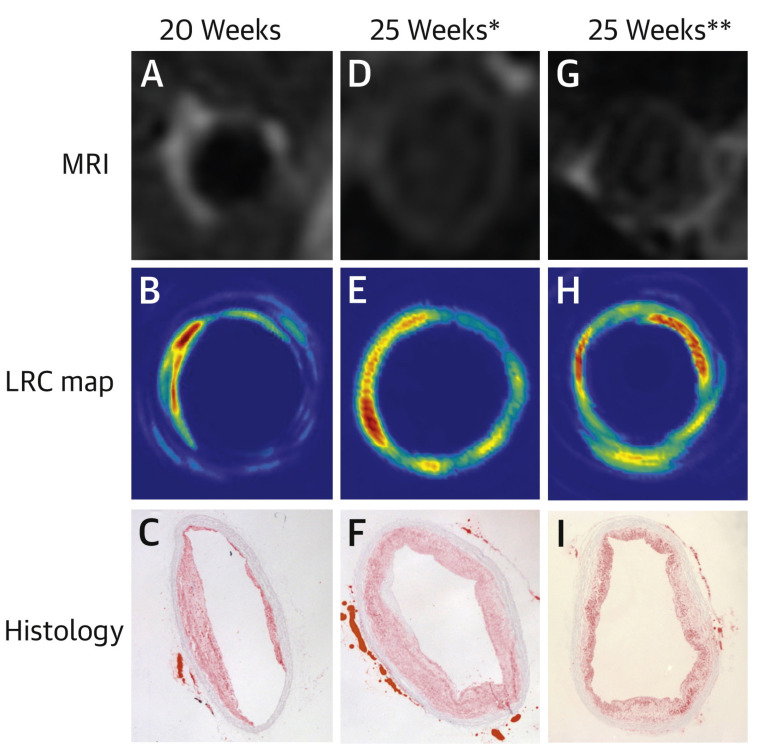
Magnetic resonance imaging (MRI) results, lipid relative concentration (LRC) map by IVPAT, and histological image of 3 rabbits in vivo examined after 20 weeks (**A**–**C**) or 25 weeks (**D**–**I**) HFC diet feeding. Reprinted from [142], Copyright (2014), with permission from Elsevier.

Intravascular PAT (IVPAT) was developed by Zhang et al. to dynamically monitor lipid concentration on blood vessel walls of rabbits fed a high fat/cholesterol (HFC) diet over a period of 20 weeks (Figure 16). Utilising a 1.8 mm diameter probe, resolution of 100 μm/380 μm axially/transversely at over 2 mm imaging depth were reported [142]. Bai et al. used a similar IVPAT system with a slimmer probe and achieved 38.1 μm/19.6 μm axially/transversely [115].

**Figure 17:**
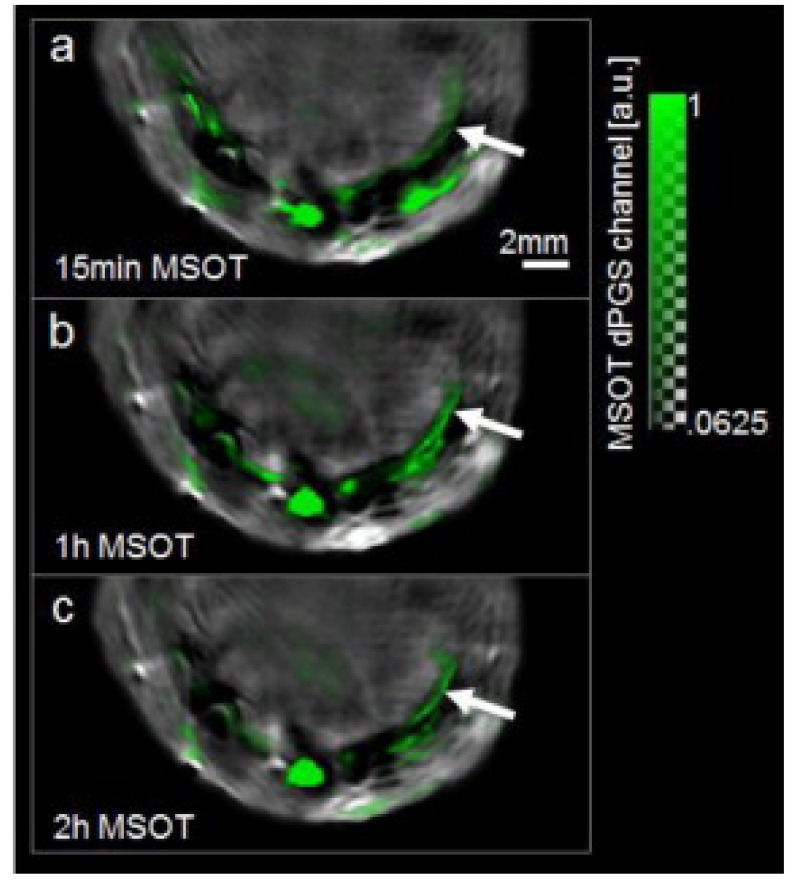
Transverse slices over time through the chest of a mouse at day 2 following myocardial infarction. (**a**) MSOT image recorded 15 min after injection showing dPGS-NIR signal superimposed in green over image taken at 900 nm. Arrow indicates site of infarction. (**b**) 1 h after injection. (**c**) 2 h after injection. Reprinted from [79], Copyright (2013), with permission from Elsevier.

MSOT, accompanied with exogenous dyes, presented non-invasive high sensitivity and resolution 2D imaging capability for myocardial infarction, when compared with other intravascular imaging techniques (Figure 17). More accurate quantitative image could be acquired with improvement of the experimental setup and enable 3D imaging [79].

### 4.3. Blood Oxygenation and Flow

Blood oxygenation levels in the skin are an important physiological measure in dermatology, cancer research and plastic surgery. A measure of blood oxygenation is the oxygen saturation of haemoglobin (sO${}_{2}$), defined as the ratio of oxy-haemoglobin concentration (CHbO${}_{2}$) to total haemoglobin concentration (CHbT). The most commonly used in vivo technique to measure blood oxygenation is near infrared spectroscopy (NIRS), but it lacks spatial resolution. This disadvantage can be solved by PAI which delivers higher spatial resolution in biological tissues.

Early techniques to determine blood oxygenation used the exponential rise of the PA signal in one-dimension [143]. However, it requires the absorbing volume to have a planar shape, which is not feasible for in vivo micro-vascular imaging. Sivaramakrishnan et al. [144] developed a quantitative PA measurement of blood oxygenation in small vessels by AR-PAM. They obtained 4% sO${}_{2}$ measurement accuracy in vitro, but much worse results (>20%) of mm measurement in rat skin micro-vasculature because of wavelength dependent light attenuation in deep tissue. Fluence compensation for accurate measurements of CHb and sO${}_{2}$ can be very challenging.

**Figure 18:**
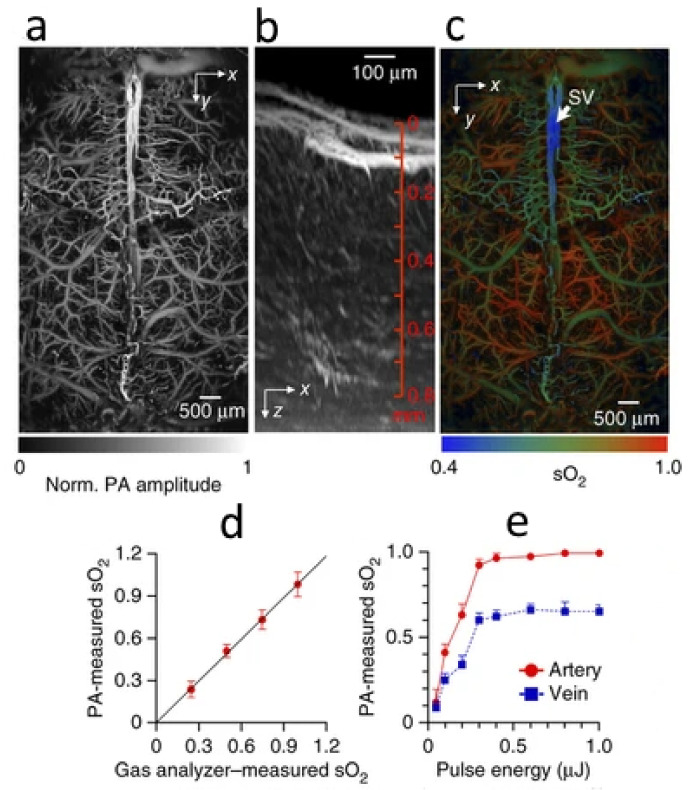
(**a**) Representative projected brain vasculature image through an intact skull image. (**b**) Representative enhanced xz projected brain vasculature image acquired over a 0.6 × 0.6 mm${}^{2}$ region with depth scanning, where the signal amplitude was normalized depthwise. (**c**) Photoacoustic microscopy of oxygen saturation of hemoglobin (sO${}_{2}$) in the same mouse brain (skull vessel) (**d**) shows the comparison of sO${}_{2}$ measurements in four blood phantoms with gas analyzer readings and (**e**) illustrates in vivo sO${}_{2}$ measurements in an artery-vein pair in a mouse ear with varied excitation pulse energies, where the data are averaged over ten measurements. Reprinted by permission from Springer Nature: Nature Methods [150] (High-speed label-free functional photoacoustic microscopy of mouse brain in action, Junjie Yao et al), COPYRIGHT (2015).

A potential solution is to incorporate PAT with diffuse reflectance spectroscopy [145] or diffuse optical tomography (DOT) [146], which can quantify the tissue’s optical properties or the fluence distribution. Alternatively, the acoustic spectra of PA signals at multiple optical wavelengths can be used to fit for absolute concentrations of Hb and sO${}_{2}$, where fluence compensation is not required [147]. Ray et al. [148] applied molecularly lifetime-based PA sensing to determine blood sO${}_{2}$. Recently, another calibration-free method for absolute sO${}_{2}$ quantification in PACT has been developed by Xia et al. based on the PA signal dynamics at different oxygenation states [149]. Yao et al. [102] combined AR-PAM with a model-based inverse reconstruction algorithm (so-called PACM) to quantify deoxygenated-Hb and sO${}_{2}$ at the small vessel level in a rodent model, where the error of the recovered absorption coefficient is less than 5%. Most recently, Yao et al. [150] implemented a single-wavelength pulse-width-based method (Figure 18) with a one-dimensional imaging rate of 100 kHz for blood oxygenation with capillary-level resolution.

By integrating fine spatial and temporal scales, PA flowoxigraphy, a new implementation of OR-PAM, has demonstrated multi-parametric imaging of oxygen released from single red blood cells (RBCs) in vivo [151]. By fast line scanning (20 Hz) along a capillary with two-wavelength excitation, PA flowoxigraphy can simultaneously measure multiple hemodynamic parameters that are required to quantify the oxygen release rate by RBCs. Experimental results show that PA flowoxigraphy can be used to image the coupling between neural activity and oxygen delivery in response to different physiological challenges, which can be useful for understanding how brain is powered at the single cell level.

### 4.4. Breast Imaging

PAI has also demonstrated the ability to map breast lesions, especially tumours. Ye et al. applied 3D-PAT with confocal laser and a 64-element linear UST array for breast cancer detection, however their study was limited to ex vivo samples only [152]. Kang et al. was able to detect real-time micro-calcification in breast which is an indicator of early-stage cancer using PAT with a linear UST array and a 690–700 nm laser using ex vivo samples [153,154]. Another prototype 3D full breast PAT used a specifically engineered UST array for optimized sensitivity and FOV designed by Xia et al. 2 mm XY resolution and 6 mm Z resolution were achieved over a target range of 170 mm × 170 mm × 170 mm [155].

As the technology became more mature, clinical studies emerged in literature. Heijblom et al. imaged 10 patients with malignancies and 2 with cysts by PA mammoscope system. PAT demonstrated higher contrast than X-rays for mapping malignancies but not cysts [156]. Li et al. reported a pilot clinical study of using PAT to image breast cancer as well as monitor neoadjuvant chemotherapy [64]. Oraevsky et al. presented a dual-mode opto-acoustic-ultrasonic tomography system for the clinical functional monitoring of breast cancer (invasive ductal carcinoma and fibroadenoma) [157]. Recently, Fakhrejahani et al. [158] gave a clinical report on a PAT system with dual illumination for breast cancer imaging. The results showed a 74.4% visibility rate (29 out of 39 cancers) at a median depth of 26.5 (3.25–51.2) mm. Moreover, it seems that age, menopausal status, body mass index, history of neoadjuvant treatment, clinical stage and histological tumor angiogenesis markers did not affect the visibility. In lumpectomy procedures, Li et al. [159] declared to achieve 75% specificity in breast tumor margin assessment by PAT, with a trade-off 100% sensitivity (only fatty tissue is considered as normal tissue). However, the specificity can be improved by analysing the frequency spectra of PA signals produced in dense connective tissue and cancer tissue with different mechanical properties.

Biofuctionalized conjugated polymer (CP) as an exogenous optical contrast agent enables molecular PA imaging for breast cancer. An MSOT setup with a 128-element concave UST array spanning an arc of 270° had a measured spatial resolution of 150–200 μm. Strong PA signals were observed after the first hour due to active targeting of the folate-CP dots to the FR+ve cancer cells while passive accumulation of the probe by the effects of enhanced permeability and retention only generated weak signals [160].

### 4.5. Urogenital Imaging

Specifically designed for endoscopic imaging, PAE is well-suited for urogenital imaging. Li et al. built an experimental PAE system with probe diameter of 12.7 mm. Ex vivo rat intestine images were acquired at a 6.25 Hz B-scan rate with axial/transverse resolution of 64 μm/105 μm and a penetration depth of 6 mm. These values were achieved by illumination with a 532 nm laser at a 5 kHz repetition rate and a UST with a central frequency of 40 MHz [112].

AR-PAM was utilized by Ding et al. for the in vivo study of mice endometriosis, an endometrial-like lesion outside Uterus endometrium, mainly on ovaries, pelvic peritoneum, rectovaginal septum and occasionally on diaphragms, pleura and the pericardium. The obtained results suggested the ability of depicting lesions and blood vessels simultaneously with a high sensitivity. Stronger PA signals were received using a 532 nm laser compared to the NIR region [161].

**Figure 19:**
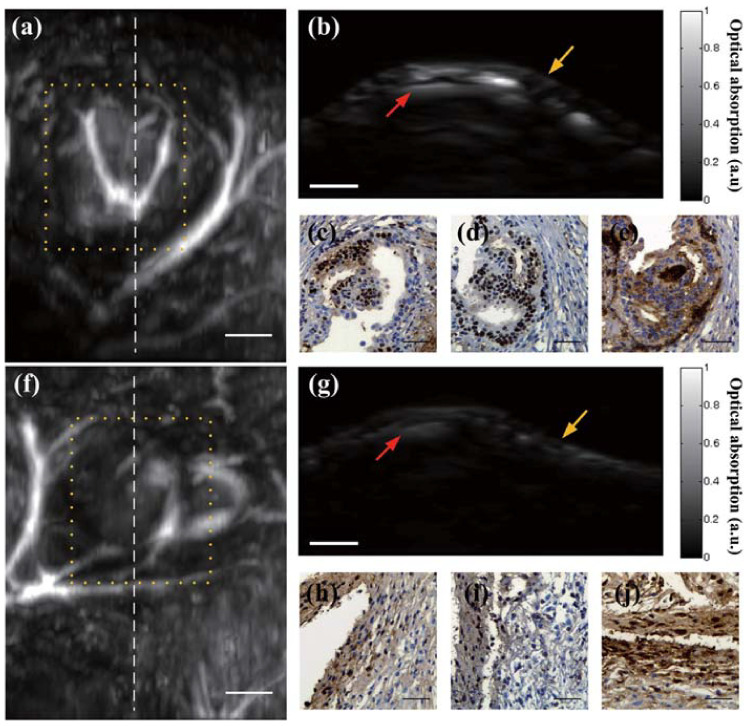
Comparison of positive and negative results of EM lesion in vivo. (**a**–**e**) Positive PAM results of EM lesion, (**a**) MAP result, (**b**) cross-sectional B-scan, corresponding to the dashed line in (**a**), (**c**) PR result, (**d**) ER result. (**e**) CD10 result. (**f**–**j**) Negative PAM results, (**f**) MAP result, (**b**) crosssectional B-scan, corresponding to dashed line in (**f**), (**c**) PR result, (**d**) ER result. (**e**) CD10 result. Scale bar: (**a**,**b**,**f**,**g**) 1 mm; (**c**,**d**,**e**,**h**,**i**,**j**) 50 mm. Reprinted from [161], Copyright (2014), with permission from John Wiley and Sons.

Ding et al. explored PA for the detection of cervical cancer to avoid conventional biopsies (Figure 19). However, the lack of in vivo studies and appropriate reconstruction algorithms prevented the quantitative analysis of efficacy [161].

### 4.6. Whole Body (Deep Tissue) Imaging

Possessing the ability to acquire in-depth target information, several PAT and MSOT systems were developed for deep-tissue and small animal whole-body imaging. Laufer et al. successfully imaged a mouse embryo in vivo using PAT with a Fabry-Perot interferometer. A Penetration depth of 10 mm can be reached with reasonable resolution [162].

**Figure 20:**
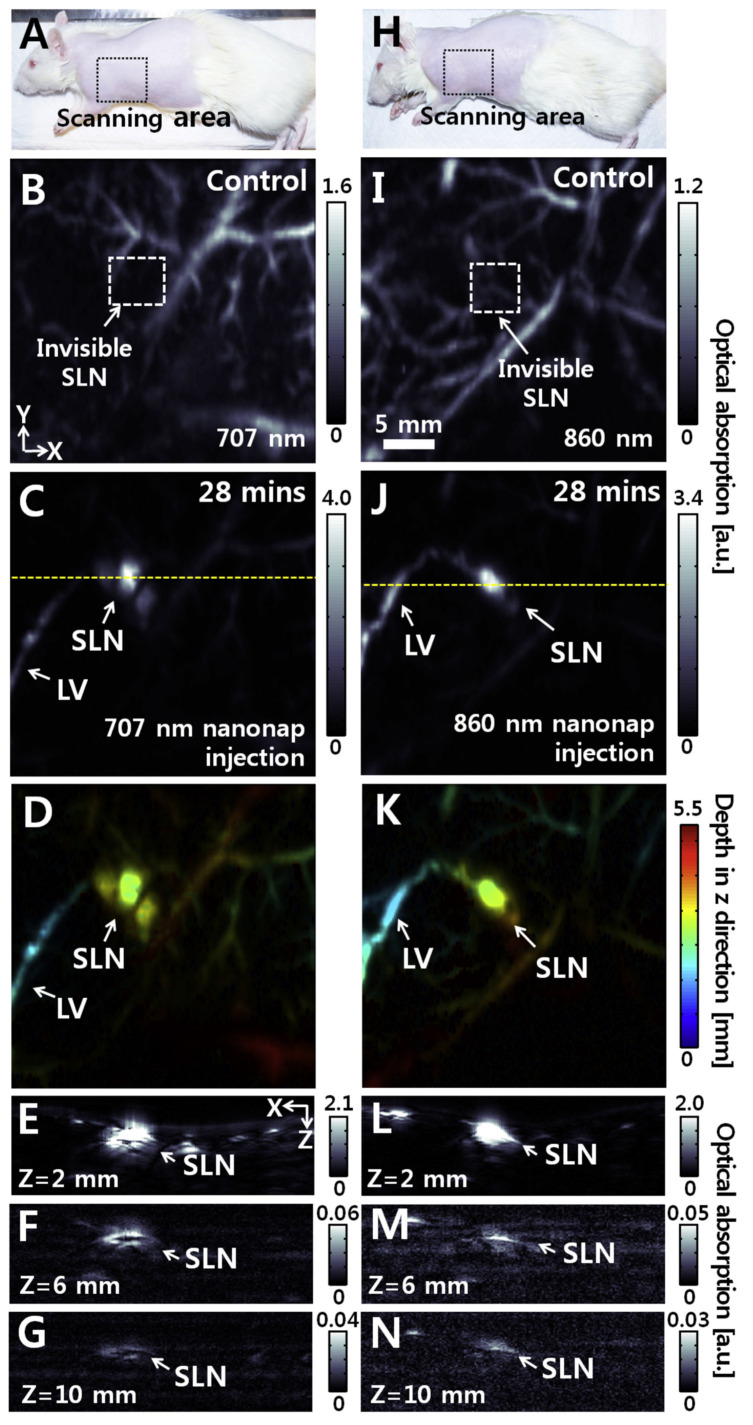
In vivo and ex vivo PA imaging of rats’ sentinel lymph nodes (SLNs) with injection of 707 nm and 860 nm nanonaps. (**A**,**H**) Photographs of the rats. (**B**,**I**) Control PA maximum amplitude projection (MAP) images acquired before injection of nanonaps. (**C**,**J**) Depth-encoded PA images obtained at 28 min post-injection of 707 nm and 860 nm nanonaps, respectively. (**D** and **K**) Depth-encoded PA images of (**C**,**J**), respectively. (**E**,**L**) Depth-resolved PA B-scan images cut along the yellow dotted lines in (**C**,**J**), respectively. (**F**,**M**) Depth-resolved PA B-scan images of the SLNs with injection of 707 nm and 860 nm nanonaps, respectively. The imaging depth was increased to 10 mm with chicken breast tissues atop the rats. (**G** and **N**) Cross-sectional PA B-scan images of the SLNs with injection of 707 nm and 860 nm nanonaps, respectively, and the PA images were acquired with two layers of chicken breast tissue atop the rats. LV: Lymphatic vessel. BV: Blood vessel. Reprinted from [166], Copyright (2015), with permission from Elsevier.

Assisted by chemical dyes as biomarkers and for contrast enhancement, targeted organ images can be acquired. For example, Morscher et al. and Taruttis et al. reported the dynamic monitoring of pharmacokinetics and biodistribution in organs such as the liver, stomach, gallbladder, vena cava , and spinal cord etc. utilizing MSOT with the circulation of ICG [76,77]. Miyata et al. employed a PAT system working in conjunction with ultrasound and fluorescence imaging to focus on hepatic malignancies in surgically re-sected liver samples [163]. Other examples uses nano-particles for optical contrast assisted MSOT or PA/fluorescence dual-mode imaging [164,165]. Genetically encoded PA contrast such as the tyrosinase-based reporter employed by Jathoul et al. provide a novel approach for PA image enhancement as well as the study of cellular and genetic processes. Catalysed by tyrosinase, the production of eumelanin contributes to a recognizable boost in PA detection by a Fabry-Perot interferometer [42]. Lee et al. [166] have used organic nano-formulated naphthalocyanines (referred to as nanonaps) for dual-color PA SLN mapping in vivo (Figure 20), in ∼10 mm deep biological tissue. Nanonaps have unique features such as higher NIR absorption than gold nanorods, no heavy metal toxicity due to its organic nature, wide spectral tuning spectral range in the NIR region, and non-shifting spectral stability at ultrahigh optical densities.

**Figure 21:**
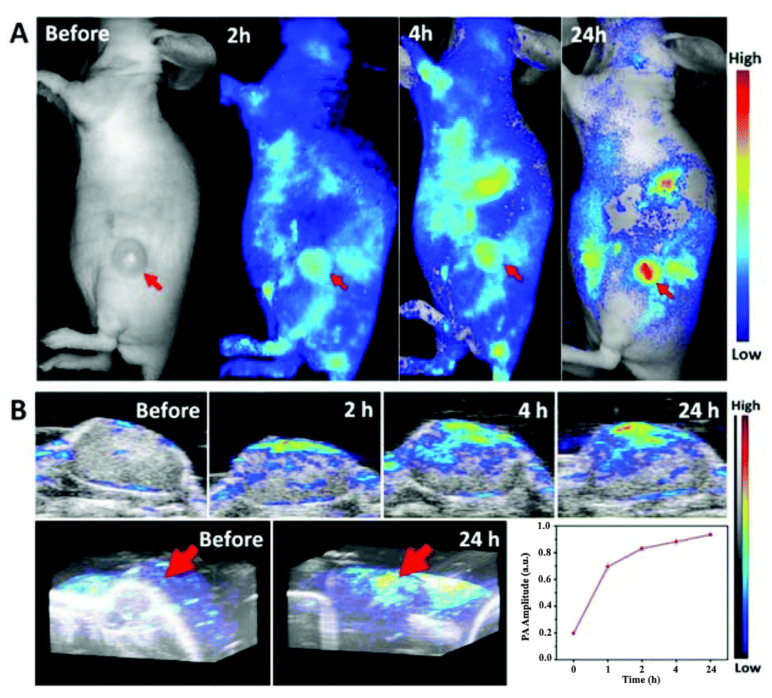
(**A**) In vivo fluorescence images and (**B**) ultrasound/PA images and quantitative analysis of PA signal of PC9 bearing mice treated with GO-PEG-DVDMS (GO-PEG 1 mg/kg, DVDMS 2 mg/kg) at 2, 4 and 24 h post-injection. Republished with permission of Royal Society of Chemistry, from [167], Copyright (2009); permission conveyed through Copyright Clearance Center, Inc.

Integrated with photo-dynamic (PDT)/photo-thermal therapy techniques, PA imaging devices can even be developed into theranostic systems. Yan et al. employed sinoporphyrin sodium (DVDMS) photosensitizer-loaded PEGylated graphene oxide (GO-PEG-DVDMS) for fluorescence/PA dual imaging (Figure 21) and PDT/PTT [167]. Sim et al. described PA-based nano-medicine for simultaneous cancer therapy and diagnosis used Human Serum Albumin NanoParticles (HSA-NPs) loaded with melanin and paclitaxel (HMP-NPs) to enhance imaging and enable treatment [168].

### 4.7. Opthalmic Imaging

US and OCT are both commonly used for ocular imaging, but PA has a higher resolution than US and greater penetration depth than OCT [169]. Zhang et al. developed photoacoustic ophthalmoscopy (PAOM) with a stationary UST contacting eyelid and a scanning laser over retina. For a 256-line B-scan a framerate of 93 Hz and an axial resolution of 23μm were achieved in 1 mm-diameter circular area FOV [170]. Another modality called PA ocular imaging was illustrated by Zerda et al. to work simultaneously with US for blood distribution in retina, choroid, and optic nerve [171]. Wu et al. improved the resolution of ocular imaging by over 10 fold using an improved focused laser delivery method [172].

**Figure 22:**
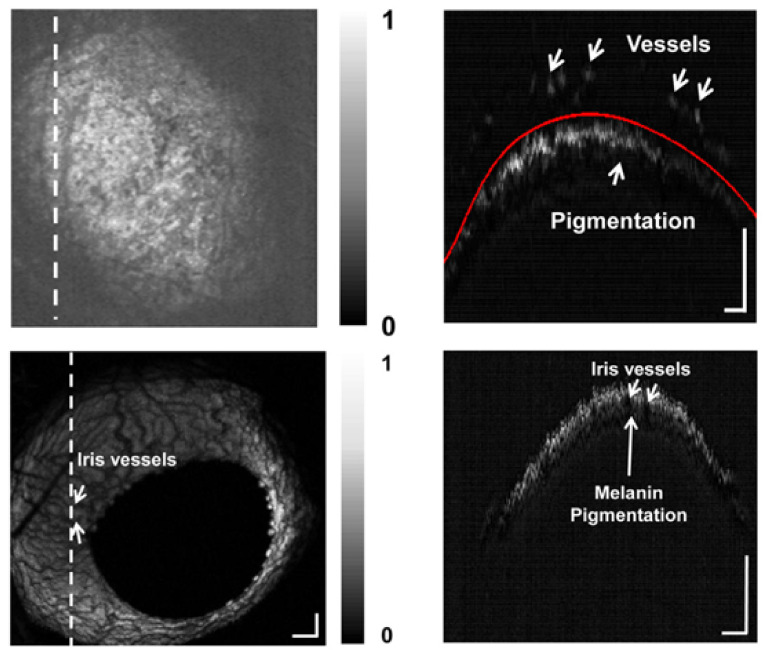
Mouse iris. Top: alkali burnt eye, MAP (**left**) and B scan of the line indicated on the left (**right**); Bottom: healthy eye. Reprinted from [173], Copyright (2014), with permission from Elsevier.

Hu et al. used spectroscopic OR-PAM to visualize the structure and sO${}_{2}$ of the iris microvasculature and related abnormalities [173]. Liu et al. successfully applied OR-PAM for in vivo corneal neovascularization (Figure 22) and were able to differentiate between alkali-burnt injured and healthy eyes [174].

### 4.8. Dental Imaging

A preliminary study of oral disease using PA was done by Li et al. suggested promising results of identifying early-stage tooth decay [175]. Hughes et al. illustrated an all-optical PAM with a path-stabilised Michelson interferometer as an ultrasonic detector of white-spot lesions at an earlier stage than current clinical techniques. The time-reversal reconstruction function of the *k*-Wave MATLAB toolbox was used for quantitative measurement of the lesion [176].

Olivi et al. presented a photon-induced PA streaming system to disinfect the root canal [177]. PA is not used as an imaging technique in this case however it provides a novel application for such phenomenon and may have the potential to be combined with PAI in the future to create devices that are more powerful and beneficial.

**Figure 23:**
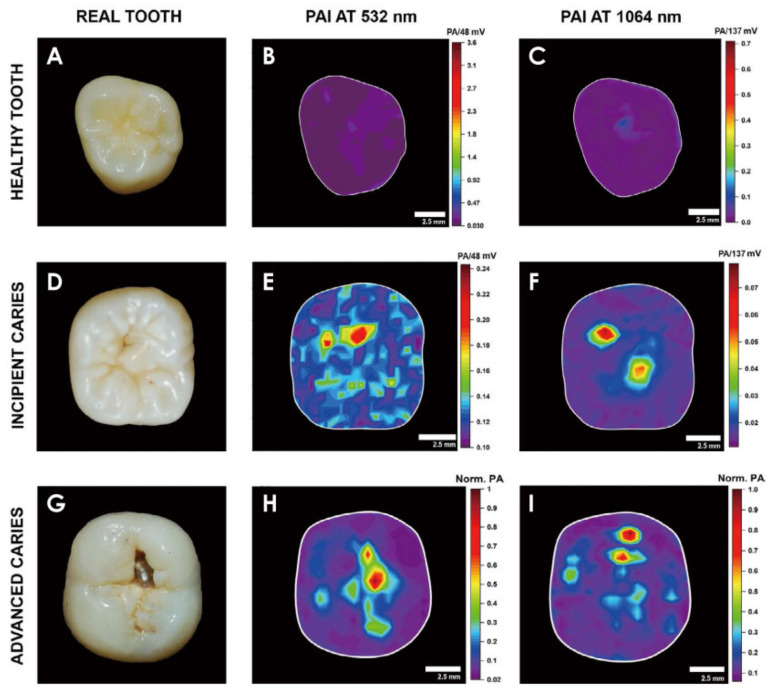
PAI of sound and carious teeth at 532 nm and 1064 nm with a 5-MHz photoacoustic detector. (**A**–**C**) Representation of the healthy tooth, incipient caries (**D**–**F**), and advanced caries (**G**–**I**) groups. Under CC BY-NC 3.0 license from [178].

Recently, Silva et al. demonstrated PAI of occlusal incipient caries [178]. It was proved that a higher intensity of the PA signal could be observed in regions with lesions, while healthy surfaces showed much less PA signal (Figure 23).

## 5. Skin Imaging

### 5.1. Molecular Diffusion/Optical Clearing

Transcutaneous vaccine delivery is a novel approach of immunization which is regarded as one of the most efficient way of fending off infectious diseases. Comparing to conventional methods, transcutaneous delivery possesses benefits of controlled-release, convenient, pain-free, and free of needle-borne infections [179]. The molecular diffusion of substances through skin layers was studied with various means to better understand the mechanism and to search for an optimised delivery method. Optical coherence tomography (OCT) has been a popular technique with a relatively higher imaging depth (up to 2 mm) [179,180], among other optical microscopies such as Raman microscopy and Two-Photon microscopy. The use of an optical clearing agent (OCA) would help improve the optical imaging depth as the diffused OCA molecules could match the refractive index of scatterers and eliminate scattering [181,182,183]. Meanwhile behaviours of diffusion could be monitored during optical clearing.

Benefitting from scalable imaging depth and the ability to image absorbers, PA imaging holds great potential of dynamically monitoring molecular diffusion. Zhou et al. applied OAC merely as an agent of imaging enhancement for their OR-PAM setup, causing a 4.4-times sensitivity increase and a more than 2.5-fold increase in resolution [184]. Liu et al. conducted similar experiments to study the optical clearing effect of a number of OCAs by observing skin samples immersed in OCA with AR-PAM [185]. Yang et al. based on Liu’s research, proposed a more dynamic monitoring of optical clearing using a combination of PA and ultrasonic imaging [186]. However, the mechanism of optical clearing and effectiveness of different OCAs are yet to be conclusive. Therefore, further investigation on this subject should be pursued.

### 5.2. Detecting Circulating Tumor Cells

**Figure 24:**
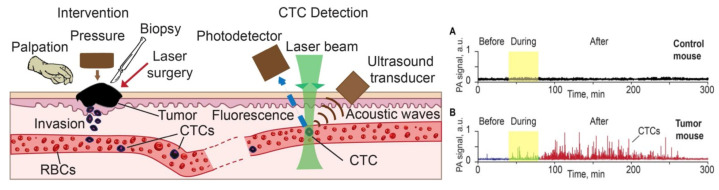
Principle of in vivo flow cytometry using PA and fluorescence methods for real-time monitoring of melanoma and breast circulating tumor cells (CTCs) directly in the bloodstream during palpation, pressure, biopsy, and surgery [187]. (Left) Schematic of different interventions. (Right) Detection methods. RBC, red blood count. (**A**) Example of PA signal trace before, during, and after pressure (120 g) on back skin and muscle of a control nude mouse (no tumor). (**B**) Example of PA signal trace before, during, and after pressure on approximately 5-mm melanoma tumor model (B16F10-green fluorescent protein [GFP]) on the nude mouse back. Reprinted from [187], Copyright (2013), with permission from John Wiley and Sons.

The cancer metastasis is the primary factor to cause death, where tumor cells release from the primary tumor and go into the blood or lymph vessels to spread throughout the body. Therefore, detecting circulating tumor cells in these vessels plays a critical role in evaluating metastasis, cancer recurrence and therapeutic efficacy [119]. However, conventional in vitro diagnosis is not sensitive enough owing to the limited volumes of blood samples. By contrast, PA flow cytometry (PAFC) has the capability to measure up to a patient’s entire blood volume in vivo (Figure 24), resulting in a significant improvement in sensitivity [122].

The major challenge of using PAFC in detecting CTCs is being able to suppress the strong PA background from the blood, which can easily mask signals from rare cell types [12]. By combining targeted nano-particles with ultra-sharp PA resonances, magnetic trapping with fiber-magnetic-PA probes, optical clearance, real-time spectral identification, and non-linear signal amplification, PAFC achieved a high sensitivity of (1 CTC/40 mL) and a throughput (up to 10 mL/min) which with existing assays is un-achievable [188].

Great progress has been made in the real-time in vivo detection of tumor cells by PAFC. Galanzha et al. recently applied Cytophone platform for the PA detection of circulating tumor cells in patients with melanoma [189]. Cytophone technology utilises an in vivo PAFC platform with a high pulse rate laser and focused ultrasound transducers for label-free detection of melanin-bearing CTCs.

The transcutaneous delivery of laser pulses via intact skin to a blood vessel results in the generation of acoustic waves from CTCs, which are amplified by vapor nano-bubbles around intrinsic melanin nano-clusters. The time-resolved detection of acoustic waves using fast signal processing algorithms makes PA data tolerant to skin pigmentation and motion. No CTC-related signals were identified in 19 healthy volunteers, but in 27 out of 28 patients with melanoma, signals were observed consistent with single, clustered, and likely rolling CTCs. The Cytophone could detect individual CTCs at a concentration of ≥1 CTC/mL in 20 s and identify clots and CTC-clot emboli. The system has great potential for in vivo blood testing with the Cytophone for early melanoma screening.

## 6. Conclusions and Prospects

PA has developed into various imaging modalities for a wide range of applications. Established reconstruction and simulation algorithms, flexibility of optical scaling, high optical contrast, high acoustic resolution and deep penetration all combine to render PA imaging advantageous over many other diagnostic modalities.

We have discussed the fundamental principles, detection techniques and major modalities including PACT, MSOT, PAM and PAE. The intensity of active research for applications targeting brain, cardiovascular, breast, whole-body/deep-tissue, ophthalmology, dentistry, molecular diffusion, blood oxygen content, and circulating tumor cells have provided great momentum to the development of the technology. PA can be applicable and rendered more viable for other applications, such as structural and functional skin imaging. Recent machine learning methods provide unique advantages when applied to the field of PAI, such as extremely fast computational time. This has great potential to facilitate clinical translation in the long term [190].

Nonetheless, some limitations still exist. Optical attenuation limits the penetration to ∼5 cm when resolutions of less than 1 mm are desired in tissues without signal averaging [2]. Ultrasound signals cannot efficiently penetrate through gas cavities or lung tissues [2]. Light propagating in tissue attains a spectrum that varies with location due to wavelength-dependent fluence attenuation, limiting the quantification accuracy of spectroscopic PA imaging [2]. Thick bones, such as the human skull, strongly attenuate incident light and ultrasonic waves. The cost of PA systems also remains one of the challenges to clinical translation [191].

PA has a range of biomedical applications and there is a growing need within the food industry [192,193]. Due to technological progress, PAI techniques continue to improve through miniaturization, usability and cost reduction [194]. As more advanced imaging techniques, contrast agents and reconstruction algorithms are developed, the benefits that PAI offers to humanity will continue to increase.

## Figures and Tables

**Table 1:** MSOT comparison with other visualization techniques, based on [84]. * E—excellent, G—good, A—average, S—satisfactory, P—poor.

	Tecnology Parameters			Application Parameters		
	Contrast *	Sensitivity	Resolution (mm)	Throughput Capacity	Easiness of Use *	Penetration Depth *
**MSOT**	E	pmol	0.05	High	E	A
**X-ray**	P	μmol	0.05	High	E	E
**XrayCT**	S	μmol	0.05	Low	G	E
**MRI**	E	nmol	0.05	Low	A	E
**US**	P	nmol	0.05	Medium	E	A
**PET**	E	fmol	1–2	Low	S	E
**SPECT**	E	fmol	1–2	Low	S	E
**Optical**	G	pmol	1–2	Medium	E	P

**Table 2:** Main features of current modalities in PA imaging.

Modality	Penetration Depth (mm)	Lateral Resolution (mm)	Axial Resolution (mm)	Application
**PACT**	50	0.7	0.7	Peripheral joints, brain, whole-body study
**MSOT**	10–50	0.15	0.5	Real-time, whole-body tomography with body navigations
**PAM**	3	0.045	0.015	Molecular or cellular imaging
**PAFC**	2–4	0.002	0.033	Circulating tumor cells detection
**PAE**	<60	0.02–0.3	0.1	Gastrointestinal or cardiovascular imaging

**Table 3:** Comparison of intravascular imaging techniques, based on [142].

Comparison of Intravascular Imaging Techniques for Characterization of Atherosclerotic Plaques				
**Physical Property**	**Technique**	**Resolution (mm)**	**Penetration (mm)**	**Specialized Lipid Imaging Capability**
Acoustic	IVUS	0.1	8–10	NA
	IVUS-RF analysis	0.1–0.2	8–10	Poor
Light scattering/absorbance	OCT	0.004–0.02	0.001–0.002	NA
	NIRS	NA	0.001–0.002	Good
Photoacoustic	IVPAT	0.1	0.002–0.004	Good

## Data Availability

Not applicable.
